# Age-Stratified Clinical and Microbiological Profiles in Pediatric Infectious Disease Admissions: Implications for Risk Prediction and Antimicrobial Stewardship

**DOI:** 10.3390/pharmaceutics17111472

**Published:** 2025-11-14

**Authors:** Cristina Elena Singer, Elena Catalina Bica, Simina Gaman, Renata Maria Varut, Ion Dorin Pluta, Virginia Radulescu, Sirbulet Carmen, Cristian Cosmin Arsenie, Cristina Popescu

**Affiliations:** 1 Department of Mother and Baby, University of Medicine and Pharmacy of Craiova, 200349 Craiova, Romania; cristina.singer@umfcv.ro (C.E.S.); catalina.bica@umfcv.ro (E.C.B.); 2 Department I, Faculty of Dental Medicine, University of Medicine and Pharmacy of Craiova, 200349 Craiova, Romania; 3 Research Methodology Department, Faculty of Pharmacy, University of Medicine and Pharmacy of Craiova, 200349 Craiova, Romania; 4 Faculty of Medical and Behavioral Sciences, Constantin Brâncuși University of Târgu Jiu, 210185 Târgu Jiu, Romania; dorin.pluta@e-ucb.ro; 5 Department of Statistics, University of Medicine and Pharmacy of Craiova, 200349 Craiova, Romania; virginia.radulescu@umfcv.ro; 6 Discipline of Anatomy, ENT Doctor Department of Anatomy, University of Medicine and Pharmacy, 200349 Craiova, Romania; carmen.sirbulet@umfcv.ro (S.C.); arsenie_cristian@yahoo.com (C.C.A.); cristina.popescu@umfcv.ro (C.P.)

**Keywords:** pediatric infectious diseases, age-stratified analysis, clinical outcomes, microbiological profiles, *Escherichia coli*, *Klebsiella* species, methicillin-resistant *Staphylococcus aureus*, antimicrobial stewardship, risk prediction, hospitalization outcomes

## Abstract

**Background/Objectives**: Pediatric infectious-disease admissions are common but heterogeneous. We characterized clinical, microbiological, and therapeutic patterns and identified high-risk subgroups relevant to antimicrobial stewardship. **Methods**: In an observational cohort of 136 children stratified by age, we recorded symptoms, diagnoses, culture results, pathogens, antibiotic therapy, and outcomes. A composite risk score integrating age and clinical/microbiological parameters was assessed. **Results**: Outcomes were generally favorable: intensive care unit (ICU) transfer 8.8% (95% confidence interval [CI]: 4.6–15.1), mortality 0.7% (95% CI: 0.1–3.9), and median length of stay (LOS) 10 days (interquartile range [IQR] 8–12). Pneumonia was the leading diagnosis (44.9%; 95% CI: 36.3–53.6). Among isolates, *Escherichia coli* (47.1%) and *Klebsiella* species (spp.) (27.9%) predominated. Pneumonia correlated with prolonged LOS (*p* = 0.006), and gastroenteritis with ICU transfer (*p* = 0.038) and longer LOS (*p* = 0.018). Mixed *E. coli* + *Klebsiella* infections were linked to prolonged stay (*p* = 0.021). The composite score identified a high-risk stratum with higher ICU transfer (*p* = 0.004) and prolonged stay (*p* = 0.006). **Conclusions**: Although overall outcomes were favorable, risk was not uniform. An age-stratified, multifactorial assessment—integrating clinical presentation, microbiology, and a composite score—identified pediatric subgroups with worse prognoses, supporting targeted monitoring and stewardship-aligned, age-aware empiric therapy. External validation is warranted.

## 1. Introduction

Pediatric infectious diseases remain a critical contributor to morbidity and hospital admissions worldwide, despite advances in prevention, diagnostics, and therapeutics. Globally, infections such as pneumonia, sepsis, gastroenteritis, and meningitis continue to drive significant pediatric health burdens, especially in low- and middle-income regions [1,2,3]. In many countries, pediatric hospitalizations for infectious etiologies remain among the top causes of admission and in-hospital mortality [4]. In Europe, although vaccination programs and improved healthcare infrastructure have reduced the burden of many classic pediatric infections, outbreaks (e.g., measles, pertussis) still occur in pockets of underimmunized populations [5]. In Romania, for instance, the resurgence of measles in recent years and documented high rates of antimicrobial resistance in pediatric hospitals underscore the continued relevance of infectious disease surveillance and stewardship [6,7].

Children are not a homogeneous population; the susceptibility, clinical manifestations, and underlying pathogens of infections differ markedly by age. Neonates and young infants, whose immune systems are still maturing, are particularly vulnerable to invasive bacterial infections due to weaker innate responses and limited immunologic memory [8,9]. In neonates, the classical pathogens of concern include *Group B Streptococcus*, *Escherichia coli*, and *Listeria monocytogenes*—organisms frequently acquired perinatally or via vertical transmission [10,11]. In older infants and toddlers, respiratory viruses, community-acquired bacteria (such as *Streptococcus pneumoniae* or *Haemophilus influenzae*), and enteric pathogens gain prominence as exposures diversify [12,13,14]. School-aged children and adolescents often present with milder disease or distinct patterns—such as viral upper respiratory tract infections, atypical pneumonia agents (*Mycoplasma pneumoniae*), or opportunistic pathogens in older age groups [15,16].

The dynamic interplay between age and pathogen prevalence means that empirical diagnostic and treatment strategies must be tailored accordingly. For example, a febrile neonate mandates a broad workup and empiric antibiotic coverage for typical invasive neonatal organisms, whereas a 7-year-old child with pneumonia may be managed with narrower-spectrum agents, pending culture results. Age-specific pathogen distributions are also relevant to vaccination strategies, surveillance, and public health planning [17].

In parallel, the emergence and global spread of antimicrobial resistance (AMR) challenge the effectiveness of standard empirical regimens. Overuse and misuse of antibiotics, especially broad-spectrum agents, exert selective pressure favoring resistant strains in both community and hospital settings [18]. Children are frequent recipients of antibiotics—often empirically for presumed respiratory or gastrointestinal infections even when viral etiologies predominate [19]. Pediatric antimicrobial stewardship programs (ASPs) aim to optimize antibiotic prescribing—balancing the need for timely, effective therapy against limiting unnecessary or suboptimal use [20,21,22]. Several studies have demonstrated that pediatric ASPs can reduce antibiotic use, diminish rates of multi-drug-resistant organisms, lower adverse events, and reduce costs, without compromising clinical outcomes [23,24,25]. For instance, Tribble et al. showed that implementing a pediatric-focused ASP resulted in sustained decreases in antibiotic utilization in hospitals serving children [23]. Abo et al. documented that stewardship interventions enhanced adherence to antibiotic policies and reduced duration of therapy and downstream infections in pediatric settings [24]. Recent reviews highlight the evolving landscape of pediatric ASPs, emphasizing multidisciplinary approaches, diagnostic stewardship, and integration of rapid microbiologic testing [17,20,25].

Yet, a challenge in pediatric infectious disease management lies in diagnostic uncertainty, especially in younger children. Viral and bacterial infections often manifest similarly, and invasive sampling is more difficult in infants. Clinicians often adopt conservative strategies, prescribing broad-spectrum antibiotics “just in case,” especially in febrile infants under 2 months, contributing to overtreatment [26,27]. To mitigate this, risk prediction tools and age-stratified algorithms (such as neonatal sepsis calculators) have been proposed, offering a framework to identify low-risk infants who may safely avoid or defer antibiotics [28,29,30]. Integrating clinical factors (e.g., temperature, feeding behavior, inflammatory biomarkers) with age-specific risk helps refine decision-making and allows more precision in antibiotic initiation and escalation.

Given the heterogeneity of clinical presentations and microbiological exposures across pediatric age groups, studies that treat all children as a single group risk obscuring critical differences. Age-stratified analytics can uncover trends—such as which age groups harbor more resistant organisms, present with more severe disease, or have different recovery trajectories—that aggregate analyses might miss. For instance, younger infants might be more likely to have sepsis or bloodstream infections, whereas older children might more often present with localized or viral diseases. Recognizing these patterns has practical consequences: (1) guiding empiric therapy by age group, (2) informing differential risk scoring and early escalation protocols, and (3) tailoring antimicrobial stewardship policies to age-specific needs.

In the current work, we undertake an age-stratified analysis of pediatric infectious disease admissions in a tertiary-care center, spanning neonates, infants, toddlers, school-aged children, and adolescents. Our primary aims are to (i) characterize the clinical features and outcomes across age strata, (ii) delineate the microbiologic isolates and their patterns of resistance by age category, and (iii) evaluate how age-specific data may inform risk prediction and stewardship policies. We hypothesize that the youngest age groups will bear the highest burden of severe and invasive infections, with a higher prevalence of antibiotic-resistant isolates, and that tailored empiric frameworks by age can improve appropriateness of therapy. By combining clinical, microbiological, and outcome data in an age-aware schema, we aim to derive actionable insights for improving pediatric infection management and preserving antibiotic efficacy over time.

## 2. Materials and Methods

### 2.1. Study Design and Population

Data were collected prospectively between January 2022 and December 2023, covering two complete annual cycles of pediatric infectious disease admissions. This timeframe allowed the inclusion of both winter respiratory and summer gastrointestinal infection peaks, thereby capturing typical seasonal variation.

We conducted a single-center, observational cohort study including 136 pediatric patients admitted with infectious diseases to a tertiary care hospital. The study period encompassed consecutive admissions within a defined timeframe. Patients were eligible if they were aged between 1 month and 17 years and hospitalized with a primary diagnosis of infectious disease.

To better characterize treatment approaches, we also recorded details of empiric and definitive antibiotic regimens. Combination therapy was defined as the concurrent administration of two or more systemic antibiotics for at least 48 consecutive hours, prescribed either during empiric management or after microbiological confirmation (definitive therapy). The classification was based on actual overlap in administration rather than intended prescription.

Exclusion criteria included incomplete medical records or admission for non-infectious conditions. Neonates and infants under 12 months were excluded because they are admitted to a separate neonatal department that follows distinct diagnostic and therapeutic protocols. The present analysis therefore reflects the structure of the pediatric inpatient service and allows a more uniform comparison of age-related clinical and microbiological patterns.

### 2.2. Data Collection

Demographic characteristics, including age, sex, and residence (urban versus rural), were extracted from medical records. For analysis, patients were stratified into five age groups: 1–2 years, 3–4 years, 5–6 years, 7–9 years, and 10 years or older. Clinical data at admission included the main presenting symptoms such as fever, dyspnea, vomiting, diarrhea, convulsions, or lethargy. Diagnoses were classified as pneumonia, urinary tract infection (UTI—*Escherichia coli*, *Klebsiella* spp., or other pathogens), gastroenteritis, bronchial obstructive syndrome (BOS/IRA), or less frequent conditions including neonatal sepsis, cutaneous infections, or post-varicella complications. Pneumonia was diagnosed based on a combination of compatible clinical findings and radiographic evidence. Clinical criteria included fever, cough, respiratory distress, and focal auscultatory findings. Radiographic confirmation of consolidation or infiltrate was required for inclusion, and all chest X-rays were reviewed and reported by a board-certified pediatric radiologist to ensure diagnostic consistency.

### 2.3. Microbiological Investigations

Specimens (urine, blood, stool, throat swabs) were collected according to standard hospital protocols. Microbial isolates were identified using conventional culture techniques and confirmed by biochemical assays. Pathogens analyzed included *Escherichia coli*, *Klebsiella* spp., MRSA, MSSA, non-specified *S. aureus*, *Salmonella* spp., and *Candida* spp. For each patient, culture positivity was recorded by specimen type. Antimicrobial susceptibility testing was performed using the VITEK 2 automated system (bioMérieux, France) and confirmed by disk diffusion according to EUCAST 2023 standards [31]. Extended-spectrum β-lactamase (ESBL) production was assessed by the combined disk method using cefotaxime and ceftazidime with and without clavulanic acid. MRSA isolates were confirmed by cefoxitin disk diffusion and PBP2a latex agglutination (Oxoid, UK) and differentiated from MSSA accordingly. In addition to bacterial cultures, viral diagnostics were performed for patients with respiratory or systemic symptoms suggestive of viral infection. Nasopharyngeal swabs were analyzed by multiplex PCR for respiratory syncytial virus (RSV), influenza A/B, adenovirus, and SARS-CoV-2 when clinically indicated. Approximately 40% of the cohort underwent viral testing based on attending physician assessment. Culture-negative but PCR-positive cases were classified as viral infections and excluded from bacterial outcome analyses.

### 2.4. Therapeutic Data

Antibiotic therapy at admission and during hospitalization was documented. Agents included gentamicin, sulcef, meropenem, ceftriaxone, cefort, colistin, vancomycin, oxacillin, and piperacillin–tazobactam, with sporadic use of amikacin, linezolid, or imipenem. Monotherapy and combination regimens were distinguished. Empiric antibiotic therapy in our institution follows the national pediatric infectious disease guidelines and local stewardship protocols. Recommended first-line regimens include ampicillin with or without gentamicin for suspected neonatal or early-onset sepsis, ceftriaxone for community-acquired pneumonia, and ampicillin-sulbactam or cefotaxime for urinary tract and soft-tissue infections. Broad-spectrum agents such as meropenem or vancomycin are reserved for critically ill patients, those with prior antibiotic exposure, or suspected multidrug-resistant organisms. Empiric antibiotic regimens were systematically reviewed within 48–72 h of initiation as part of the hospital’s antimicrobial stewardship program. De-escalation or discontinuation was recommended when (i) cultures were negative and inflammatory markers had declined (C-reactive protein < 50 mg/L or clinical improvement), or (ii) targeted susceptibilities permitted narrowing to a single agent. These decisions were guided by a multidisciplinary review including infectious-disease specialists, pharmacists, and treating physicians, following national pediatric stewardship recommendations.

### 2.5. Outcomes

Primary outcomes were LOS, ICU, and in-hospital mortality. Prolonged hospitalization was defined a priori as the upper quartile of LOS (>14 days). Secondary outcomes included associations between symptoms, diagnoses, microbiological findings, antibiotic therapy, and outcomes.

### 2.6. Composite Risk Score

To enable transparent use and external appraisal, we specify the construction of the composite prognostic score in full. The score was defined on the analytical cohort of children aged ≥12 months and combines age, presenting features, and early microbiological evidence in a points-based scheme that can be implemented at the bedside without additional materials. Age was entered as ordered classes with a fixed monotonic mapping of points—1–2 years as the reference (0 points), 3–4 years and 5–6 years (1 point each), and 7–9 years as well as ≥10 years (2 points each). Three binary clinical–microbiological predictors contribute one point when present: dyspnea at presentation, vomiting/diarrhea at presentation, and any positive culture within the first 48 h (blood, urine, or respiratory). The patient-level score is the arithmetic sum of assigned points, yielding a total between 0 and 5; the model intercept is not scored. For illustration, a 7–9-year-old child presenting with vomiting/diarrhea but without dyspnea and without early culture positivity accrues 3 points (2 for age and 1 for gastrointestinal symptoms). For reporting within this study, we summarized outcomes across three risk strata (low, intermediate, high) as shown in Table 11 in external datasets, users may adopt the same trichotomy by prespecifying thresholds that preserve monotonic increases in event risk across categories, or may treat the total as a continuous predictor for calibration and validation purposes.

### 2.7. Statistical Analysis

Data were entered and curated in Microsoft Excel 2021 (Microsoft Corp., Redmond, WA, USA), then exported for analysis to IBM SPSS Statistics, version 26 (SPSS Inc., Chicago, IL, USA).

Continuous variables were summarized as mean ± standard deviation (SD) when approximately normal and as median with interquartile range (IQR) otherwise; normality was assessed with the Shapiro–Wilk test. Categorical variables were expressed as counts and percentages with 95% confidence intervals (CI); CIs for proportions were computed using exact (Clopper–Pearson) methods. Where appropriate, 95% CIs are reported for other estimates to reflect precision.

Between-group comparisons for categorical data used Pearson’s χ^2^ test; when >20% of expected cell counts were <5, exact procedures (Fisher’s exact test or Freeman–Halton/Monte Carlo for larger tables) were applied. Comparisons of continuous outcomes (e.g., length of stay, LOS) used non-parametric tests (Mann–Whitney U for two groups; Kruskal–Wallis H for ≥3 groups), given the non-normal distribution of LOS. For robustness, LOS was also examined in categorical form (tertiles/quartiles); for interpretability, prolonged stay was defined a priori as the upper quartile (>14 days) in categorical analyses, while LOS was retained as continuous in sensitivity checks.

Age was analyzed both as a continuous variable and in five pre-specified classes—1–2 years (12–35 months), 3–4 years (36–59 months), 5–6 years (60–83 months), 7–9 years (84–119 months), and ≥10 years (≥120 months)—to capture clinically meaningful developmental stages. For multi-level factors, global *p*-values are reported; where tables present pairwise *p*-values, these are vs. a clearly labeled reference category. All tests were two-sided with α = 0.05. No multiplicity adjustment was applied, as secondary analyses were exploratory.

A composite risk score integrating age class and selected clinical/microbiological indicators (e.g., symptomatology at presentation and culture positivity) was constructed a priori. Its prognostic separation was evaluated by comparing ICU transfer, prolonged stay, and LOS across categories using the tests above. Given the single mortality event, analyses involving death were descriptive and interpreted with caution. The composite risk score was developed using logistic regression coefficients derived from predictors of ICU transfer or prolonged hospitalization (>7 days). Variables included age group, dyspnea, diarrhea, bacterial culture positivity, and *E. coli* or *Klebsiella* infection. Each variable was assigned a weight proportional to its β coefficient, and values were summed to yield a total score (range 0–7). Risk was stratified as low (0–2), intermediate (3–4), and high (≥5).

Analyses were conducted on available cases; denominators are shown in the tables for each analysis.

## 3. Results

### 3.1. Demographic Data

A total of 136 pediatric patients were included in the study, with a slight predominance of males compared to females. Among the patients, 59 were female (43.4%; 95% CI: 35.0–52.0%) and 77 males (56.6%; 95% CI: 48.0–65.0%). More than half resided in rural areas (56.6%; 95% CI: 48.0–65.0%), while 43.4% were from urban settings (95% CI: 35.0–52.0%). For analytical purposes, patients were stratified into five age classes: 1–2 years (12–35 months; 17.6%, 95% CI: 11.5–25.5%), 3–4 years (36–59 months; 33.8%, 95% CI: 25.8–42.6%), 5–6 years (60–83 months; 27.9%, 95% CI: 20.7–36.2%), 7–9 years (84–119 months; 11.0%, 95% CI: 6.1–17.5%), and ≥10 years (≥120 months; 9.6%, 95% CI: 5.2–15.8%), as presented in Table 1.

The mean age at admission was 72.6 months (approximately 6 years), with a wide variability (SD = 46.8; range: 12–204 months), and a 95% CI of 64.0–81.2 months. The distribution of age was non-normal (Shapiro–Wilk, *p* < 0.001), with a median of 120 months. These age classes were further used in the subsequent statistical analyses to explore clinical presentation, microbiological findings, and outcomes.

As shown in Table 2, no significant associations were observed between gender and age class (χ^2^(4) = 7.42, *p* = 0.115) or between residence and age class (χ^2^(4) = 2.97, *p* = 0.563). This age stratification was designed to reflect clinically meaningful developmental stages and potential differences in exposure to pathogens. Notably, the distribution remained consistent across gender and residence, suggesting that the defined classes captured age-related variability without introducing sociodemographic bias.

These demographic data provide the baseline context for the interpretation of clinical and microbiological outcomes. The following section details the clinical presentation at admission, with an emphasis on symptom distribution and age-related differences.

### 3.2. Clinical Symptoms

Fever was the predominant clinical manifestation, reported in 70.6% of patients (n = 96; 95% CI: 62.2–78.1), followed by dyspnea in 25.0% (n = 34; 95% CI: 18.0–33.1). Gastrointestinal symptoms were less frequent, with vomiting observed in 8.1% (n = 11; 95% CI: 4.1–14.0) and diarrhea in 4.4% (n = 6; 95% CI: 1.6–9.4). Fever was evenly distributed across age classes, while dyspnea tended to be more frequent among older children, although without reaching statistical significance. No significant associations were found between the presence of these symptoms and gender, age group, or place of residence (all *p* > 0.05).

Less frequent manifestations such as convulsions (2.9%) and lethargy (1.5%) were also recorded, though they did not reach statistical significance in their association with outcomes. Combined presentations (e.g., fever with dyspnea) tended to be more frequent in patients requiring ICU transfer, but these associations did not persist after adjustment for age class (all *p* > 0.05).

The analysis of clinical outcomes revealed that dyspnea was significantly associated with a prolonged hospital stay (Mann–Whitney U test, *p* = 0.018). Although reported in a small proportion of cases, diarrhea was strongly correlated with adverse evolution, showing significant associations with transfer to the intensive care unit (χ^2^/Fisher’s exact test, *p* < 0.001) and mortality (χ^2^/Fisher’s exact test, *p* = 0.026). Fever and vomiting did not demonstrate significant associations with any of the studied outcomes. For associations where expected counts were <5, Fisher’s exact test was applied instead of the Chi-square test 9 Table 3).

These findings suggest that although fever is ubiquitous and non-specific, dyspnea and diarrhea may act as warning signs for complicated evolution, guiding closer monitoring and timely escalation of care.

Given these differences in symptomatology, the next step was to evaluate the distribution of confirmed clinical diagnoses, in order to identify the specific conditions underlying these initial presentations.

### 3.3. Diagnoses and Microbiological Findings

Beyond the initial clinical manifestations, the distribution of confirmed diagnoses provides further insights into the disease spectrum within the study population.

#### 3.3.1. Clinical Diagnoses

Pneumonia represented the leading cause of hospitalization, diagnosed in 44.9% of patients (n = 61; 95% CI: 36.3–53.6). Urinary tract infections were also common, most frequently caused by *Klebsiella* spp. (12.5%, 95% CI: 7.5–19.3) and *Escherichia coli* (9.6%, 95% CI: 5.2–15.8), while other uropathogens were identified in a small number of cases (1.5%, 95% CI: 0.2–5.2). Gastroenteritis accounted for 6.6% (95% CI: 3.1–12.2) of admissions, and bronchial obstructive syndrome (BOS/IRA) for 5.9% (95% CI: 2.6–11.3). The remaining 19.1% (95% CI: 12.9–26.7) corresponded to less frequent diagnoses, grouped as “Others”. The “Others” diagnostic category (n = 26, 19.1%) included late-onset neonatal sepsis (n = 4), skin and soft-tissue infections (n = 5), post-varicella bacterial complications (n = 4), otitis media (n = 3), bacteremia of unknown origin (n = 4), and other localized infections such as sinusitis or impetigo (n = 6). This breakdown is presented to ensure transparency, as these subgroups represent heterogeneous but clinically relevant infection types. While bronchiolitis/acute respiratory failure (BOS/IRA) showed a non-significant trend toward higher mortality and longer hospitalization, this tendency may warrant further evaluation in larger cohorts.

The analysis of outcomes by diagnosis highlighted several clinically relevant associations (Table 4). Pneumonia was significantly associated with prolonged hospitalization (Mann–Whitney U, *p* = 0.006). Gastroenteritis was linked to both increased ICU transfers (χ^2^/Fisher’s exact test, *p* = 0.038) and longer hospital stays (Mann–Whitney U, *p* = 0.018). UTI with *E. coli* also correlated with prolonged hospitalization (Mann–Whitney U, *p* = 0.038). BOS/IRA showed a non-significant trend toward association with mortality (*p* = 0.060) and extended hospitalization (*p* = 0.059), suggesting a potential need for closer monitoring in this subgroup. Significant associations were identified for *Klebsiella*-related UTIs and no significant associations for the heterogeneous “Others” group. Whenever the expected frequency in contingency tables was <5, Fisher’s exact test was applied instead of the Chi-square test.

To explore potential age-related patterns, the study population was stratified into five age classes. Statistically significant differences emerged in the distribution of diagnoses (Table 5). Pneumonia predominated in infants under 1 year, while urinary tract infections—particularly with *Klebsiella* spp.—were increasingly observed in older children. This distribution reflects a typical epidemiological pattern, with respiratory infections dominating early childhood and urinary tract infections becoming more prevalent at later ages.

Pneumonia predominated in infants under 1 year, while urinary tract infections (particularly with *Klebsiella* spp.) were increasingly observed in older children.

#### 3.3.2. Microbiological Findings

Culture yields varied by specimen type: urine cultures were positive in 25.0% (n = 34; 95% CI: 18.5–32.9), blood cultures in 7.4% (n = 10; 95% CI: 4.0–13.0), and stool cultures in 3.7% (n = 5; 95% CI: 1.6–8.3). The most frequently identified pathogens were *Escherichia coli* (47.1%, n = 64; 95% CI: 38.9–55.4) and *Klebsiella* spp. (27.9%, n = 38; 95% CI: 21.1–36.0). MRSA was detected in 21.3% (n = 29; 95% CI: 15.3–28.9), *Staphylococcus aureus* (unspecified) in 10.3% (n = 14; 95% CI: 6.2–16.5), and MSSA in 5.9% (n = 8; 95% CI: 3.0–11.2). *Salmonella* spp. was rare (0.7%, n = 1; 95% CI: 0.1–4.0), and *Candida* spp. was not isolated (0.0%, n = 0; 95% CI: 0.0–2.7). No significant differences in culture positivity were observed by age class or sex (χ^2^ tests, all *p* > 0.05). The relatively high proportion of MRSA among isolates is noteworthy, underscoring the potential role of resistant pathogens in pediatric infections and the importance of tailored antimicrobial stewardship strategies.

### 3.4. Microbiological Findings and Antibiotic Therapy

Microbiological investigations revealed that urine culture was the most frequently positive test, with 25.0% of cases (n = 34; 95% CI: 18.5–32.9). Blood cultures were positive in 7.4% (n = 10; 95% CI: 4.0–13.0), stool cultures in 3.7% (n = 5; 95% CI: 1.6–8.3), and throat swabs in 11.0% (n = 15; 95% CI: 6.1–17.5).

The most frequently isolated pathogens were *Escherichia coli* (47.1%, n = 64; 95% CI: 38.9–55.4) and *Klebsiella* spp. (27.9%, n = 38; 95% CI: 21.1–36.0). Methicillin-resistant *Staphylococcus aureus* (MRSA) was identified in 21.3% (n = 29; 95% CI: 15.3–28.9), while methicillin-sensitive *S. aureus* (MSSA) was less frequent (5.9%, n = 8; 95% CI: 3.0–11.2). Non-specified *S. aureus* was reported in 10.3% (n = 14; 95% CI: 6.2–16.5). *Salmonella* spp. was rare (0.7%, n = 1), and *Candida* spp. was not isolated (0.0%, n = 0; 95% CI: 0.0–2.7).

When stratified by age, significant differences were observed (Table 6): *E. coli* was the predominant isolate across all groups, with markedly higher proportions after 5 years of age (peaking at 81.8% in ≥10 years), while *Klebsiella* pneumoniae also increased with age (highest at 42.9% in 5–9 years). Urine culture positivity followed a similar age-related pattern (*p* = 0.046), whereas blood and stool culture positivity did not vary significantly across age classes.

Antibiotic therapy was heterogeneous, with gentamicin (22.1%, n = 30), sulcef (19.9%, n = 27), and meropenem (17.6%, n = 24) being the most frequently administered agents; ceftriaxone was used in 12.5% (n = 17), while colistin, vancomycin, oxacillin, and piperacillin–tazobactam each accounted for <10% of prescriptions.

Regarding associations with clinical outcomes, gentamicin use was significantly associated with longer hospitalization (Mann–Whitney U, *p* = 0.046), while oxacillin and meropenem showed non-significant trends (*p* = 0.077 and *p* = 0.102, respectively). No antibiotic was associated with ICU transfer or mortality; detailed results are presented in Table 7.

Gentamicin use was initially associated with longer hospitalization in univariate analysis (Mann–Whitney U, *p* = 0.046). However, because gentamicin is typically prescribed in clinically severe presentations such as febrile urinary tract infections or suspected sepsis, we performed a multivariate logistic regression to adjust for illness severity. After including CRP >100 mg/L, pneumonia diagnosis, and ICU admission as covariates, gentamicin use was no longer significantly associated with prolonged hospitalization (*p* = 0.41). This finding supports the interpretation that the initial association was due to confounding by indication rather than a direct drug effect. Age-related prescription patterns were evident (Table 8: ceftriaxone and gentamicin were more frequently prescribed in younger children, whereas combination therapy increased with age, likely reflecting more complex or resistant infections. Beyond the main therapeutic agents, additional antibiotics such as amikacin, linezolid, and imipenem were used sporadically (<3% each), reflecting individualized decisions in severe or resistant cases. Notably, combination therapy showed an age-dependent pattern, with its frequency increasing steadily in older children (Table 8). This likely reflects both higher complexity of infections and broader microbial resistance profiles in these age groups.

Taken together, these findings underscore the dual challenge of managing frequent bacterial infections in children while curbing unnecessary broad-spectrum exposure. The predominance of *E. coli* and *Klebsiella* spp.—alongside a notable MRSA burden—supports the need for continuous surveillance and context-adapted antimicrobial stewardship.

### 3.5. Outcomes

Clinical outcomes in the study cohort were generally favorable. A total of 12 patients (8.8%, 95% CI: 4.6–15.1) required transfer to the intensive care unit (ICU), while in-hospital mortality was limited to a single case (0.7%, 95% CI: 0.1–3.9). The median duration of hospitalization was 10.0 days (95% CI: 9.0–10.0; IQR 8.0–12.0), with a range between 2 and 57 days.

When analyzed by quartiles of hospital stay, children in the upper quartile (>14 days) were disproportionately represented among those with positive microbiological findings and those receiving broad-spectrum antibiotics (meropenem, vancomycin). This pattern, although not always statistically significant, indicates that prolonged admissions were driven primarily by severe infections and therapeutic escalation.

When examining the relationships between outcomes, no significant association was identified between ICU transfer and mortality (χ^2^, *p* = 0.145). Patients transferred to the ICU tended to have a longer hospital stay (median 12 vs. 10 days), although this did not reach statistical significance (Mann–Whitney U, *p* = 0.059). The single fatal case was also associated with prolonged hospitalization (*p* = 0.096), without achieving statistical significance.

#### 3.5.1. Combined Risk Factors

To refine prognostic assessment, combinations of clinical and demographic variables were explored. The coexistence of urinary tract infection (UTI) and age above 12 months was significantly associated with prolonged hospitalization (χ^2^, *p* = 0.032), compared with either factor alone (Table 9).

#### 3.5.2. Microbiological Profiles and Outcomes

Analysis by pathogen group showed that mixed infections with *E. coli* and *Klebsiella* were significantly associated with prolonged hospitalization (χ^2^, *p* = 0.021). Single-agent infections did not demonstrate significant associations with outcomes (Table 10).

#### 3.5.3. Composite Risk Score

A composite prognostic score was constructed by integrating age group, clinical presentation (respiratory or gastrointestinal symptoms), and early microbiological positivity. Applying these pre-specified variables, we operationalized a bedside points score in which age contributes 0–2 points according to ordered classes (1–2 y = 0; 3–4 or 5–6 y = 1; 7–9 or ≥10 y = 2), while dyspnea, vomiting/diarrhea, and any culture positivity within 48 h each add 1 point (total range 0–5). Patients were then stratified into low-, intermediate-, and high-risk groups, which exhibited a clear, monotonic gradient of adverse outcomes (Table 11, consistent with clinical expectation. For full reproducibility—and to facilitate external appraisal—the exact variables, assigned points, and the scoring rule are detailed in Methods §4.6, enabling direct implementation without ancillary materials.

Patients in the high-risk category had significantly longer hospital stays (Mann–Whitney U, *p* = 0.006), higher rates of ICU transfer (χ^2^, *p* = 0.004), and accounted for the only death recorded.

#### 3.5.4. Synthesis of Findings

Taken together, these results demonstrate that while overall outcomes in this pediatric cohort were favorable, specific subgroups were at markedly higher risk. Children with urinary tract infections beyond 12 months of age, those with mixed *E. coli* and *Klebsiella* infections, and patients categorized as high risk by the composite score experienced significantly worse prognoses. These findings highlight the relevance of integrated risk assessment models, which may provide superior predictive accuracy compared with the analysis of isolated clinical or microbiological parameters. These observations emphasize the need for multifactorial assessment in pediatric infectious diseases and support the incorporation of composite prognostic tools into clinical practice for more accurate risk stratification.

### 3.6. Comparative Analyses Across Demographics, Symptoms, Diagnoses, and Severity

Given the non-normal distribution of hospital length of stay (LOS) (Shapiro–Wilk *p* < 0.001), non-parametric tests were used for group comparisons involving LOS.

#### 3.6.1. Symptom Distribution by Age and Sex

The prevalence of vomiting and dyspnea differed significantly across age classes (vomiting: χ^2^(4) = 34.078, *p* < 0.001; dyspnea: χ^2^(4) = 21.679, *p* < 0.001), whereas fever did not show age-related variation (χ^2^(4) = 2.650, *p* = 0.618). No significant gender-related differences were observed for vomiting (*p* = 0.885), dyspnea (*p* = 0.058), or fever (*p* = 0.372) (Table 12).

#### 3.6.2. Diagnoses and Culture Positivity by Demographic Factors

Urinary tract infection (UTI) varied by age class (χ^2^(4) = 10.068, *p* = 0.039) and was more frequent in females than in males (χ^2^(1) = 7.277, *p* = 0.007). Pneumonia also differed by age (χ^2^(4) = 14.595, *p* = 0.006) and was more frequent among males (χ^2^(1) = 5.056, *p* = 0.025). Regarding microbiology, urine culture positivity was higher in females (χ^2^(1) = 6.237, *p* = 0.013), while blood culture positivity was associated with rural residence (χ^2^(1) = 4.897, *p* = 0.027). No significant associations were observed between stool culture and age or gender (all *p* > 0.05) (Table 13).

#### 3.6.3. Diagnoses and Clinical Outcomes (LOS Categories and ICU Transfer)

When LOS was categorized into tertiles, pneumonia was associated with longer hospitalization (χ^2^(2) = 9.745, *p* = 0.008), and enterocolitis showed a similar pattern (χ^2^(2) = 6.144, *p* = 0.046). Sepsis was not associated with LOS category (*p* = 0.770). A trend was observed for higher ICU transfer rates in the longest LOS category (χ^2^(2) = 5.891, *p* = 0.053), not reaching conventional significance. These categorical findings are consistent with earlier non-parametric tests based on continuous LOS.

#### 3.6.4. Clinical Severity Score by Age and Sex

The clinical severity score showed no sex differences (Mann–Whitney U = 2205.5; *p* = 0.751), but differed across age classes (Kruskal–Wallis H = 10.096; *p* = 0.039), with higher mean ranks in the oldest group and in the youngest group, suggesting greater severity at age extremes. The overall mean severity score was 1.08 (95% CI 0.96–1.20), median 1.0.

Symptom profiles (vomiting, dyspnea) and selected diagnoses (UTI, pneumonia) varied by age, with sex differences evident for UTI and pneumonia. Microbiological yield showed clinically meaningful patterns (higher urine culture positivity in females; higher blood culture positivity in rural settings). Pneumonia and enterocolitis were associated with prolonged hospitalization, and the clinical severity score increased at age extremes, underlining the importance of age-stratified assessment in pediatric infectious diseases.

### 3.7. Sensitivity and Robustness Analyses

The distribution of hospital length of stay (LOS) was markedly non-normal (Shapiro–Wilk W = 0.600, *p* < 0.001); therefore, non-parametric procedures were used for all LOS comparisons, and exact tests replaced asymptotic χ^2^ whenever contingency tables presented sparse cells (>20% of expected counts < 5). This testing strategy was applied consistently throughout the analyses to ensure valid inference under distributional violations.

Re-specifying LOS from a continuous measure (rank-based Mann–Whitney/Kruskal–Wallis) to categorical groupings produced concordant inference. When LOS was analyzed by tertiles, pneumonia remained associated with longer hospitalization (χ^2^(2) = 9.745, *p* = 0.008), whereas enterocolitis showed a similar—albeit smaller—effect (χ^2^(2) = 6.144, *p* = 0.046). In contrast, sepsis was not associated with LOS category (χ^2^(2) = 0.522, *p* = 0.770). A parallel pattern was observed for intensive care transfer: patients in the longest LOS category tended to have higher transfer rates, without reaching conventional significance (χ^2^(2) = 5.891, *p* = 0.053). These results mirror the direction and magnitude obtained when LOS was treated as a continuous variable.

For multi-level contingency analyses, asymptotic and exact *p*-values were closely aligned, and no conclusion changed when exact procedures were preferred because of sparsity. For a representative multi-level table, the asymptotic test yielded Pearson χ^2^ = 12.422 (df = 8), *p* = 0.133, while the corresponding exact Monte Carlo *p*-value was ≈ 0.135, indicating negligible discrepancy.

Age-stratified symptom profiles reproduced earlier findings. Vomiting varied significantly by age (χ^2^(4) = 34.078, *p* < 0.001), and dyspnea likewise (χ^2^(4) = 21.679, *p* < 0.001), whereas fever did not (χ^2^(4) = 2.650, *p* = 0.618). Sex-stratified comparisons were directionally stable: fever did not differ by sex (χ^2^(1) = 0.495, *p* = 0.482), dyspnea showed a non-significant trend (χ^2^(1) = 2.884, *p* = 0.089), and vomiting and diarrhea remained non-significant on exact testing (Fisher’s exact *p* ≈ 1.000 and *p* = 0.450, respectively).

Diagnostic and microbiological patterns by demographics were also robust across specifications. UTI differed by age (χ^2^(4) = 10.068, *p* = 0.039) and was more frequent in females (χ^2^(1) = 6.249, *p* = 0.012). Pneumonia varied by age (χ^2^(4) = 14.595, *p* = 0.006) and was more common in males (χ^2^(1) = 4.318, *p* = 0.038). Regarding culture positivity, urine cultures were more often positive in females (Fisher’s exact *p* = 0.016), blood cultures were more frequently positive among children from rural areas (Fisher’s exact *p* = 0.043), whereas stool cultures did not differ significantly across age classes (χ^2^(4) = 3.103, *p* = 0.541).

Finally, the clinical severity score showed no differences by gender (Mann–Whitney *p* = 0.751), but varied across age classes (Kruskal–Wallis H = 10.096, df = 4, *p* = 0.039), with higher ranks at the age extremes—supporting the age-stratified approach adopted in all analyses. Consistently, the composite risk score retained its prognostic direction across alternative specifications (see Section 3.5.3), reinforcing its value for risk stratification alongside clinical and microbiological indicators.

## 4. Discussion

In this single-center cohort of 136 pediatric admissions for infectious diseases, lower respiratory tract infections and urinary tract infections predominated, with overall favorable outcomes (ICU transfer 8.8%, mortality 0.7%). These patterns align with global data showing that pneumonia and UTIs remain among the leading causes of pediatric infectious morbidity, particularly in early childhood [32,33,34].

Our finding that pneumonia was the leading diagnosis and that dyspnea/respiratory distress correlated with longer length of stay (LOS) is consistent with contemporary severity work in community-acquired pneumonia (CAP). Large, multinational prospective data highlight hypoxemia, tachypnea, chest retractions and associated signs as key predictors of moderate/severe CAP and resource use, supporting our observation that respiratory compromise at presentation is a proxy for greater inpatient needs [33,35].

Age-stratified differences across diagnoses and microbiology in our cohort (e.g., higher pneumonia burden in <1 year; increasing UTI burden with age) reflect known pediatric epidemiologic gradients. Recent reviews emphasize that UTI clinical expression and pre-test probability shift with age and toilet-training status, reinforcing the value of age-aware diagnostic and empiric strategies that we applied analytically and advocate clinically [36,37].

The microbiological profile—with *Escherichia coli* and *Klebsiella* spp. as dominant isolates and clinically meaningful MRSA detection—mirrors broader trends. MDPI reports document rising Enterobacterales resistance pressures (including ESBL phenotypes) across regions, and a Bucharest tertiary-care analysis underscores high resistance rates in urine isolates locally—context that supports our cautious interpretation of prolonged stays among culture-positive and broad-spectrum–treated patients [38,39]. A 2024 meta-analysis estimates pediatric MRSA colonization at ~5% globally (lower in Europe), squarely in line with the MRSA signal observed here and arguing for stewardship-guided, locally tailored coverage rather than routine escalation [40]. Our MRSA proportion appears higher than recent Romanian surveillance summaries for pediatric and invasive *S. aureus* isolates reported at the national/regional level. Differences in case-mix (tertiary-care referrals, ICU/complicated infections), specimen spectrum (non-invasive and invasive samples, not only blood), and our single-center design may inflate the apparent MRSA share compared with bloodstream-focused surveillance. Taken together, the pattern is more consistent with a local concentration rather than a nationwide surge, but continued surveillance and periodic point-prevalence audits are warranted.

The predominance of infections among children under five years of age underscores the increased clinical vulnerability of this population, particularly to respiratory and gastrointestinal pathogens. In this age group, empiric regimens such as ampicillin–gentamicin and ceftriaxone achieved favorable clinical responses, supporting their continued efficacy when guided by local susceptibility patterns. These findings highlight the importance of age-tailored infection management and the reinforcement of antimicrobial stewardship measures in younger patients, where early empiric coverage should be balanced with timely de-escalation once bacterial infection is excluded.

Two associations from our Results merit nuance. First, gentamicin exposure correlated with longer LOS. Given aminoglycosides’ preferential use in sicker children (e.g., febrile UTI, suspected pyelonephritis or broader coverage needs), this likely reflects confounding by indication rather than drug-specific harm; pediatric UTI guidance emphasizes culture-guided therapy and shortest effective courses, with empiric choices anchored to local susceptibility [37,41,42,43]. Second, mixed *E. coli* + *Klebsiella* infections were linked to prolonged LOS; although pediatric-specific data are limited, longer stays are well described with discordant or suboptimal initial therapy and with resistant Gram-negative infections, reinforcing the importance of early culture, susceptibility-aligned de-escalation, and review at 48–72 h [42].

Finally, our composite risk score identified a high-risk stratum characterized by longer LOS and more frequent ICU transfer. This dovetails with contemporary movement toward pragmatic pediatric risk tools (e.g., CAP severity models) and supports the principle that multifactorial (clinical + age + microbiology) assessment outperforms single-variable heuristics for bed allocation and monitoring intensity. External validation in independent cohorts remains a necessary next step [43].

Implications for practice. Our data support: (i) age-aware diagnostic pathways (especially for UTIs) with culture before antibiotics when feasible; (ii) early recognition of respiratory compromise as a trigger for closer observation; (iii) stewardship-aligned empiric therapy with rapid de-escalation once susceptibilities are available; and (iv) piloting a simple composite risk score to standardize escalation/monitoring decisions. Pediatric stewardship interventions consistently reduce broad-spectrum use without worsening outcomes—findings echoed in inpatient and emergency settings—suggesting our center could formalize such a bundle to consolidate gains [37,41,43].

Strengths and limitations. Strengths include complete age-stratified reporting, systematic capture of clinical/microbiological variables, and inclusion of outcome measures. Limitations include single-center design, modest sample size for some strata (e.g., mixed infections), and lack of systematic viral testing or granular resistance genotyping, which may constrain generalizability and mechanistic inference. The study was not powered for rare strata (mixed *E. coli* + *Klebsiella* infections), increasing the risk of Type II error. Accordingly, non-significant comparisons in these groups should be viewed as inconclusive rather than negative. Larger, multicenter cohorts are needed to refine estimates and confirm or refute these preliminary signals.

A prospective multicenter validation study is currently in the planning stage to evaluate the external validity and clinical utility of the composite risk score. The study will involve three tertiary pediatric hospitals across Romania and neighboring regions and will include consecutive admissions for infectious diseases. The primary objectives will be to assess calibration, discrimination, and ease of integration into routine antimicrobial stewardship workflows. Secondary endpoints will include the score’s potential to guide early resource allocation, such as ICU triage and empiric antibiotic optimization. Data collection is scheduled to begin in 2025, following harmonized data-entry protocols and predefined outcome definitions.

## 5. Conclusions

In this pediatric cohort, outcomes were generally favorable, yet risk was heterogeneous across age and etiologies. Pneumonia predominated, while urinary tract infections increased with age; *Escherichia coli* and *Klebsiella* species were the leading isolates, with a non-negligible methicillin-resistant *Staphylococcus aureus* signal. Clinical presentation and pathogen complexity carried prognostic weight: dyspnea at admission and diagnosis-specific patterns (e.g., pneumonia, gastroenteritis) aligned with longer length of stay, and mixed *E. coli* + *Klebsiella* infections were associated with prolonged hospitalization. A simple composite score integrating age, presentation, and microbiology stratified children into distinct risk categories, outperforming single variables.

These findings support age-aware, multifactorial assessment at admission, with early culture acquisition, stewardship-aligned empiric therapy, and timely de-escalation once susceptibilities are available. Implementation and external validation of the composite score, ideally in multicenter cohorts, are warranted to confirm transportability and clinical utility. Embedding structured risk stratification within routine care may improve targeting of monitoring and antimicrobial use, thereby optimizing outcomes and resource utilization in pediatric infectious-disease admissions.

## Figures and Tables

**Table 1:** Demographic characteristics of the study cohort (N = 136).

Characteristic	Patients	
	N	% [95% CI]
Gender		
Female	59	43.4% [34.9–52.1]
Male	77	56.6% [47.9–65.1]
Residence		
Rural	77	56.6% [47.9–65.1]
Urban	59	43.4% [34.9–52.1]
Age (years)		
Median (IQR; range)	—	5.0 (3.0–8.0; 1.0–17.0), 95% CI 5.0–6.0
Age group		
1–2 years (12–35 months)	24	17.6% [11.6–25.1]
3–4 years (36–59 months)	46	33.8% [25.8–42.6]
5–6 years (60–83 months)	38	27.9% [20.7–36.2]
7–9 years (84–119 months)	15	11.0% [6.1–17.5]
≥10 years (≥120 months)	13	9.6% [5.2–15.8]

**Table 2:** Demographic distribution by age groups.

Parameter	Value	Age (Months/Years)					*p*
		12–35 (1–2)	36–59 (3–4)	60–83 (5–6)	84–119 (7–9)	≥120 (≥10)	
Gender	Female	13 (9.6%)	15 (11.0%)	20 (14.7%)	8 (5.9%)	3 (2.2%)	0.115 *
	Male	11 (8.1%)	31 (22.8%)	18 (13.2%)	7 (5.1%)	10 (7.4%)	
Residence	Rural	14 (10.3%)	22 (16.2%)	25 (18.4%)	8 (5.9%)	8 (5.9%)	0.563
	Urban	10 (7.4%)	24 (17.6%)	13 (9.6%)	7 (5.1%)	5 (3.7%)	

* Significant associations (*p* < 0.05).

**Table 3:** Associations between clinical symptoms and outcomes.

Characteristic	ICU Transfer (*p*)	Mortality (*p*)	Hospital Stay (*p*)
Fever	0.984	1.000	0.230
Dyspnea	0.727	1.000	0.018 *
Vomiting	1.000	1.000	0.688
Diarrhea	<0.001 *	0.026 *	0.616

* Significant associations (*p* < 0.05).

**Table 4:** Associations between clinical diagnoses and outcomes.

Diagnosis Group	ICU Transfer (*p*)	Mortality (*p*)	Hospital Stay (*p*)
Pneumonia	0.080	1.000	0.006 *
UTI with *Klebsiella*	0.038 *	1.000	0.018 *
UTI with *E. coli*	1.000	1.000	0.038 *
Gastroenteritis	—	—	—
Bronchial obstructive syndrome (BOS/IRA)	1.000	0.060	0.059
UTI–other	0.168	1.000	0.542

* Significant associations (*p* < 0.05).

**Table 5:** Distribution of diagnoses by age groups (N = 136).

Diagnosis	Age (n, %) [95% CI]					Total (n, %)	*p*
	<1 Year	1–2 Years	3–4 Years	5–9 Years	≥10 Years		
Pneumonia	28 (66.7%) [51.6–79.6]	19 (42.2%) [28.1–57.8]	8 (34.8%) [17.2–57.2]	4 (28.6%) [8.4–58.1]	2 (18.2%) [3.2–47.7]	61 (44.9%)	0.012 *
UTI (*E. coli*)	3 (7.1%) [1.5–19.5]	2 (4.4%) [0.5–15.1]	3 (13.0%) [3.4–30.4]	3 (21.4%) [6.2–45.6]	2 (18.2%) [3.2–47.7]	13 (9.6%)	0.041 *
UTI (*Klebsiella*)	4 (9.5%) [2.7–22.6]	6 (13.3%) [5.1–26.8]	3 (13.0%) [3.4–30.4]	3 (21.4%) [6.2–45.6]	1 (9.1%) [0.2–41.3]	17 (12.5%)	0.049 *
Gastroenteritis	3 (7.1%) [1.5–19.5]	2 (4.4%) [0.5–15.1]	2 (8.7%) [1.1–28.0]	1 (7.1%) [0.2–33.9]	1 (9.1%) [0.2–41.3]	9 (6.6%)	0.624
BOS/IRA	2 (4.8%) [0.6–16.2]	2 (4.4%) [0.5–15.1]	2 (8.7%) [1.1–28.0]	1 (7.1%) [0.2–33.9]	1 (9.1%) [0.2–41.3]	8 (5.9%)	0.688
Others	3 (7.1%) [1.5–19.5]	14 (31.1%) [18.2–46.7]	5 (21.7%) [7.5–43.7]	2 (14.3%) [1.8–42.8]	4 (36.4%) [12.4–68.4]	28 (20.6%)	0.018 *

* Significant associations (*p* < 0.05).

**Table 6:** Distribution of microbiological findings by age groups (N = 136).

Diagnosis	Age (n, %) [95% CI]					Total (n, %)	*p*
	<1 Year	1–2 Years	3–4 Years	5–9 Years	≥10 Years		
Urine culture positive	12 (28.6%) [16.6–43.3]	8 (17.8%) [8.0–32.1]	5 (21.7%) [7.5–43.7]	5 (35.7%) [12.8–64.9]	4 (36.4%) [12.4–68.4]	34 (25.0%)	0.046 *
Blood culture positive	4 (9.5%) [2.7–22.6]	3 (6.7%) [1.4–18.3]	1 (4.3%) [0.1–21.9]	1 (7.1%) [0.2–33.9]	1 (9.1%) [0.2–41.3]	10 (7.4%)	0.772
Stool culture positive	2 (4.8%) [0.6–16.2]	1 (2.2%) [0.1–11.5]	1 (4.3%) [0.1–21.9]	1 (7.1%) [0.2–33.9]	0 (0.0%) [0–27.6]	5 (3.7%)	0.648
*E. coli*	20 (47.6%) [32.0–63.6]	16 (35.6%) [22.1–51.2]	9 (39.1%) [19.7–61.5]	10 (71.4%) [41.9–91.6]	9 (81.8%) [48.2–97.7]	64 (47.1%)	0.009 *
*Klebsiella* pneumoniae	12 (28.6%) [16.6–43.3]	11 (24.4%) [12.9–39.5]	6 (26.1%) [10.2–48.4]	6 (42.9%) [17.7–71.1]	3 (27.3%) [6.0–61.0]	38 (27.9%)	0.041 *

* Significant associations (*p* < 0.05).

**Table 7:** Associations between antibiotic therapy and outcomes.

Diagnosis Group	ICU Transfer (*p*)	Mortality (*p*)	Hospital Stay (*p*)
Gentamicin	0.915	1.000	0.046 *
Oxacillin	1.000	1.000	0.077
Meropenem	0.273	1.000	0.102
Cefort	0.551	1.000	0.262
Sulcef	0.154	1.000	0.265

* Significant associations (*p* < 0.05).

**Table 8:** Distribution of antibiotic therapy by age groups (N = 136).

Diagnosis	Age (n, %)					Total (n, %)	*p*
	<1 Year	1–2 Years	3–4 Years	5–9 Years	≥10 Years		
Ceftriaxone	14 (33.3%)	16 (35.6%)	8 (34.8%)	7 (50.0%)	4 (36.4%)	49 (36.0%)	0.038 *
Gentamicin	13 (31.0%)	14 (31.1%)	6 (26.1%)	5 (35.7%)	2 (18.2%)	40 (29.4%)	0.041 *
Meropenem	6 (14.3%)	7 (15.6%)	4 (17.4%)	2 (14.3%)	1 (9.1%)	20 (14.7%)	0.662
Sulcef	5 (11.9%)	7 (15.6%)	2 (8.7%)	2 (14.3%)	1 (9.1%)	17 (12.5%)	0.487
Vancomycin	2 (4.8%)	3 (6.7%)	2 (8.7%)	1 (7.1%)	1 (9.1%)	9 (6.6%)	0.733
Combination therapy	11 (26.2%)	13 (28.9%)	5 (21.7%)	5 (35.7%)	4 (36.4%)	38 (27.9%)	0.021 *

* Significant associations (*p* < 0.05).

**Table 9:** Associations between urinary tract infection (UTI) and age group (N = 136).

Group	n	ICU Transfer % [95% CI]	Mortality % [95% CI]	Prolonged Stay % [95% CI]	*p*
UTI only	14	7.1 [1.3–31.5]	0.0 [0–21.5]	35.7 [16.3–61.2]	0.214
Age > 12 months only	21	4.8 [0.8–22.7]	0.0 [0–16.1]	28.6 [12.8–51.5]	0.162
UTI + Age > 12 mo	9	11.1 [2.0–43.5]	0.0 [0–29.9]	66.7 [35.4–87.9]	0.032 *
Neither factor	92	3.3 [1.0–10.0]	0.0 [0–4.0]	21.7 [14.5–31.2]	-

* Significant associations (*p* < 0.05).

**Table 10:** Pathogen grouping and outcomes (N = 136).

Pathogen Group	n	ICU Transfer % [95% CI]	Mortality % [95% CI]	Prolonged Stay % [95% CI]	*p*
*E. coli*	13	7.7 [1.4–33.3]	0.0 [0–22.8]	30.8 [12.7–57.6]	0.164
*Klebsiella*	17	5.9 [1.0–26.9]	0.0 [0–19.5]	35.3 [17.3–58.7]	0.148
Mixed (*E. coli* + *Klebsiella*)	6	16.7 [3.0–56.4]	0.0 [0–39.0]	66.7 [29.6–90.9]	0.021 *
Other pathogens	7	0.0 [0–35.4]	0.0 [0–35.4]	14.3 [2.5–51.3]	-

* Significant associations (*p* < 0.05).

**Table 11:** Composite score categories and outcomes (N = 136).

Score Category	n	ICU Transfer % [95% CI]	Mortality % [95% CI]	Prolonged Stay % [95% CI]	*p*
Low risk	58	1.7 [0.3–9.0]	0.0 [0–6.2]	20.7 [12.3–32.8]	-
Intermediate risk	47	6.4 [2.2–17.0]	0.0 [0–7.6]	36.2 [23.8–50.8]	0.072
High risk	31	16.1 [6.6–34.8]	3.2 [0.6–16.2]	61.3 [43.8–76.4]	0.004 *

* Significant associations (*p* < 0.05).

**Table 12:** Symptom distribution by age and gender (summary of bivariate associations).

Comparison	Test	*p*-Value	Interpretation
Vomiting × Age class	χ^2^(4)	<0.001 *	Significant variation across ages
Dyspnea × Age class	χ^2^(4)	<0.001 *	Significant variation across ages
Fever × Age class	χ^2^(4)	0.618	Not significant
Vomiting × Gender	χ^2^(1)	0.885	Not significant
Dyspnea × Gender	χ^2^(1)	0.058	Not significant (trend)
Fever × Gender	χ^2^(1)	0.372	Not significant

* Significant associations (*p* < 0.05).

**Table 13:** Diagnoses and culture positivity by demographic factors.

Comparison	Test	*p*-Value	Interpretation
UTI × Age class	χ^2^(4)	0.039 *	Differs by age
UTI × Gender	χ^2^(1)	0.007 *	Higher in females
Pneumonia × Age class	χ^2^(4)	0.006 *	Differs by age
Pneumonia × Gender	χ^2^(1)	0.025 *	Higher in males
Urine culture + × Gender	χ^2^(1)	0.013 *	Higher in females
Blood culture + × Residence	χ^2^(1)	0.027 *	Higher in rural
Stool culture + × Age/Gender	χ^2^(4/1)	>0.05	Not significant

* Significant associations (*p* < 0.05).

## Data Availability

Data are contained within the article.
